# In situ behavioral responses of crustacean zooplankton to an approaching seismic survey

**DOI:** 10.1038/s41598-025-20568-8

**Published:** 2025-10-13

**Authors:** Saskia Kühn, Emilie Hernes Vereide, Jonas Bousquet, Karen de Jong, Katja Heubel, Anne Christine Utne-Palm

**Affiliations:** 1 Coastal Ecology, Research and Technology Center West Coast, Kiel University, Kiel, Germany; 2 Biodiversity and Environmental Impact, DNV, Oslo, Norway; 3 Acoustics and Observation Methodologies, Institute of Marine Research, Bergen, Norway; 4 Fisheries, Institute of Marine Research, Bergen, Norway

**Keywords:** *Calanus finmarchicus*, Copepods, Impulsive noise, Underwater noise, Swimming speeds, Escape behavior, Biological techniques, Ecology, Ecology, Environmental sciences, Ocean sciences

## Abstract

The impacts of underwater noise from seismic surveys on zooplankton remain poorly understood despite their critical ecological role. This study investigated the effects of in situ airgun shots on the swimming behavior of the copepod *Calanus finmarchicus* at distances from over 4000 to less than 100 m from the seismic airgun array (3060 in^3^, 50.1 L). Copepods were deployed in a cage equipped with a stereo camera system to track individual swimming behavior. Our findings reveal significant changes in swimming speeds and speed-based behavioral classifications: *Swimming*, *Sinking*, and *Jumping*. During airgun exposure, the swimming speed increased significantly, displaying a quadratic relationship around an airgun shot. More copepods jumped, with higher relative *jumping* counts per individual, following a non-linear relationship with distance from the seismic source. *Sinking* duration decreased, while *swimming* lasted longer during shoot periods. Furthermore, our findings suggest that changes in fluid flow speeds and low-frequency sound induced by airgun shots may have driven some of the observed responses, underscoring the complex interaction between seismic activity and copepod behavior. This study not only sheds light on the behavioral effects of impulsive noise on pelagic copepods but also introduces a novel methodology for field research involving small aquatic organisms.

**Supplementary Information:**

The online version contains supplementary material available at 10.1038/s41598-025-20568-8.

## Introduction

Copepods, particularly the genus *Calanus* spp., are among the most abundant metazoans in the ocean, play a pivotal role in transferring carbon energy from primary producers to higher trophic levels, support the biological pump, and serve as essential prey for commercially important marine species^1–3^. One stressor of increasing concern to this group are marine seismic surveys^4,5^. The majority of seismic surveys, conducted to explore sub-seafloor oil and gas deposits, use impulsive sound waves generated by airgun array shootings. These sound waves penetrate the seabed and reflect back to receivers; analysis of their travel times allows geophysicists to generate detailed images of geological formations. Typically carried out by the energy sector, seismic surveys produce impulsive sound that dominates soundscapes spatially and temporally, spanning several thousand square kilometers and persist several months^6,7^. The rapid release of air from an airgun generates a high-amplitude, high-pressure broadband sound, with most energy concentrated in low frequencies^7–10^. The low-frequency sound propagates efficiently through the ocean^6^, making seismic surveys a well-recognized source of risks to marine life, nearby and over considerable distances. Effects have been predominantly documented in non-zooplanktonic taxa including marine mammals and fish. Close to the source, the intense sound levels—reaching up to 260 dB rms re 1 µPa at 1 m and 200 dB re 1 µPa²s SEL^8–10^—can cause immediate physical harm, such as hearing damage or auditory shifts, posing survival risks to marine animals^11^. Further from the source, the sound can still have widespread effects, such as masking important communication signals^6,12^ and altering behavior across large areas, with impacts observed several kilometers away^7,13^. Behavioral disturbances can disrupt critical activities such as feeding, mating, and habitat use. For example, impulsive sounds have been shown to alter activity patterns in fish and marine mammals^14,15^, interfere with mating calls in cetaceans and other species, and lead to the avoidance^16–18^ of essential habitats for resting and breeding. However, responses can vary, with some studies reporting weak or no effects, like in spawning Atlantic cod (*Gadus morhua*), which did not abandon their spawning site in response to far-distant seismic airgun exposure but some individuals swam deeper^19,20^. In general, behavioral changes can signal distress and reduce fitness over time, resulting in cumulative energy deficits, stress, or lower reproductive success^11,14^.

Increasing research on anthropogenic underwater sound shows complex, species-specific effects on zooplankton ranging from neutral to negative impacts^5^ such as mortality and developmental delays. Fields et al.^21^ reported limited mortality in *Calanus sp.* following seismic airgun exposure, whereas McCauley et al.^4^ observed significantly higher mortality rates. Similarly, Vereide et al.^22,23^ documented increased mortality and developmental delays in *Acartia tonsa* nauplii^23^, and effects varied by species^22^. These discrepancies may stem from zooplankton size, species susceptibility to diverse exposure conditions such as acoustic intensity and airgun-induced fluid motion (increased fluid motion measured in Fields et al.^21^. However, behavioral research is limited: one lab study observed changes under airgun-like pressure drops^22^, while another reported no significant behavioral effects from in situ airgun exposure^21^.

Copepods can trigger cascading effects throughout ecosystems when their behavior is disrupted^24–26^. They exhibit diverse behaviors—feeding, diel vertical migration, mating, and anti-predator responses — that are sensitive to environmental stressors. Global warming and plastic pollution, for example, are well-known to affect these behaviors^27,28^. Acoustic pollution is increasingly recognized as another disruptive factor^5^. Behavioral responses often serve as early indicators of stress or environmental change, while slower physiological, morphological, and genetic responses reflect longer-term adaptations^29^.

To the best of our knowledge, no prior studies have conducted in situ close-view video recordings to observe the behavior of individual copepods under seismic exposure at sea. Most in situ studies of zooplankton behavior, including copepods, have relied on larger-scale techniques such as active acoustic monitoring^30,31^. Our study fills this gap by providing data on individual zooplankton movements, complementing existing methods and informing on zooplankton behavior under impulsive noise.

The zooplankton species studied in this experiment was the ecologically and commercially important copepod *Calanus finmarchicus.* Although the mechanisms underlying seismic survey impacts on zooplankton remain unclear^5^, potential effects include physical damage^21,22^ and sensory disruption, which can alter swimming orientation^4^. *C. finmarchicus* detects environmental stimuli through mechanoreceptors on its antennae and body surface, sensitive to water movement and, to some extent, pressure^32,33^. Airgun sound waves produce hydrodynamic changes that could be perceived or potentially injure copepods^22^. Therefore, we predict that exposure will alter the species’ swimming speeds^22^ and behavior^34,35^ around a commercial seismic airgun survey.

## Results

The following results present sound levels, copepod swimming speeds and behaviors, and hydrodynamic conditions measured at progressively decreasing distances between an approaching seismic vessel’s airgun array and the stationary research vessel conducting the experiments (Fig. 6). Copepods were filmed within submerged mesh cages (Fig. 7), allowing for detailed observations of free-swimming behavior during in situ airgun exposure and control conditions without shooting.

### Sound recordings

**Fig. 1:**
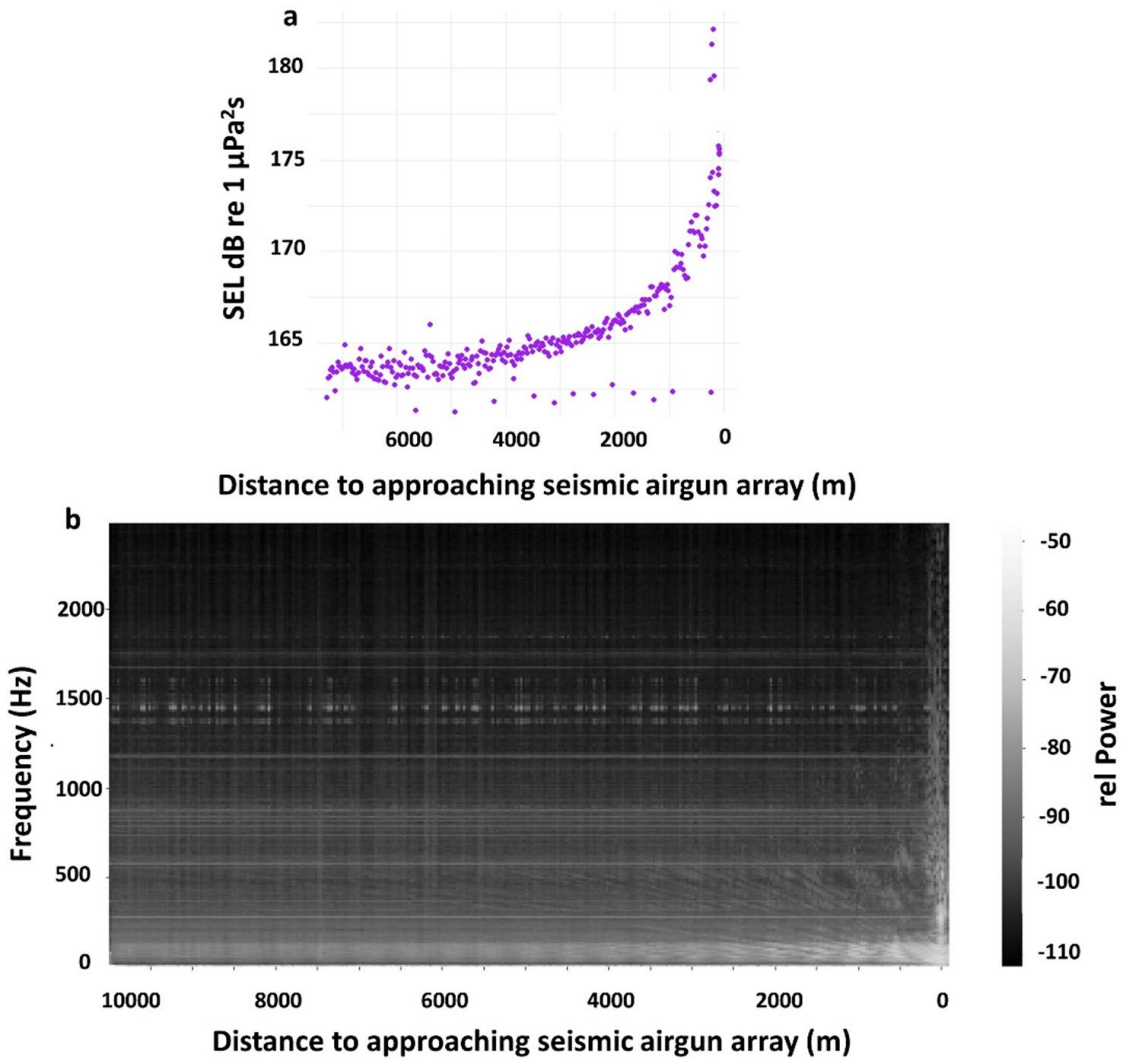
Airgun Sound with distance. (**a**) Calibrated sound exposure levels (SEL) in dB re 1 µPa²·s (y-axis), computed from time-domain pressure recordings using 10-second windows (~ 10.7 s between airgun shots), are plotted against distance from the airgun array. (**b**) Spectrogram with distance. The x-axis represents different recording distances from the airgun array, while the y-axis shows frequencies up to 2500 Hz. The grey scale indicates power in dB, computed from spectrogram power levels. Data recorded on 5th of May.

On 5th of May, the hydrophone recorded a maximum broadband sound exposure level (SEL) of 182 dB re 1 µPa²s (Fig. 1a) and a peak-to-peak pressure level of up to 35 kPa^30^. These measurements were taken as the seismic vessel passed, with the airgun array approaching within 80 m of the research vessel. SEL increased significantly with decreasing distance, following a logarithmic trend of + 3.02 dB per tenfold distance reduction (log model: *n* = 303, t = −36.72, *p* < 0.001; Fig. 1a). Ambient SEL refers to the sound exposure level measured during periods when seismic airgun shooting was below background noise levels— dominated by the research vessel—with an average SEL of 158 ± 0.5 dB re 1 µPa^2^s^30^. Below 1000 Hz - sound levels increased at distances less than 2500 m from the airgun array (Fig. 1b).

### Behavior categories

Three behaviors were identified and based on relative speed data categorized; *Jumping*,* Sinking*, and *active Swimming*.

A sample of *Jumping* (*n* = 11) and *Sinking* (*n* = 8) behaviors was manually extracted to calculate relative speeds (Δ*v*). For *Jumping*, Δ*v*_jump_ speeds ranged from minimum − 22 mm s^−1^ to maximum 839 mm s^−1^, with the first quartile at 75 mm s^−1^ and the third quartile at 274 mm s^−1^ (median = 190 mm s^−1^, mean = 201 mm s^−1^). For *Sinking*, Δ*v*_float_ speeds ranged from − 144 mm s^−1^ to 126 mm s^−1^, with the first quartile at −25 mm s^−1^ and the third quartile at 14 mm s^−1^ (median = −5 mm s^−1^, mean = −5.45 mm s^−1^). For categorization, we used the third quartile of *Jumping* speeds and the interquartile range of *Sinking* speeds. Additionally, for the *Sinking* category, we added an angle of 90° for directional categorization, that falls within the interquartile range of observed sinking angles (< Q3 119°), capturing the highest concentration of observed cases aligned with flow-directed movement. *Swimming* behavior is categorized as being less than the first quartile and greater than the third quartile of Δ*v*_float_, but less than the third quartile Δ*v*_jump_.

**Fig. 2:**
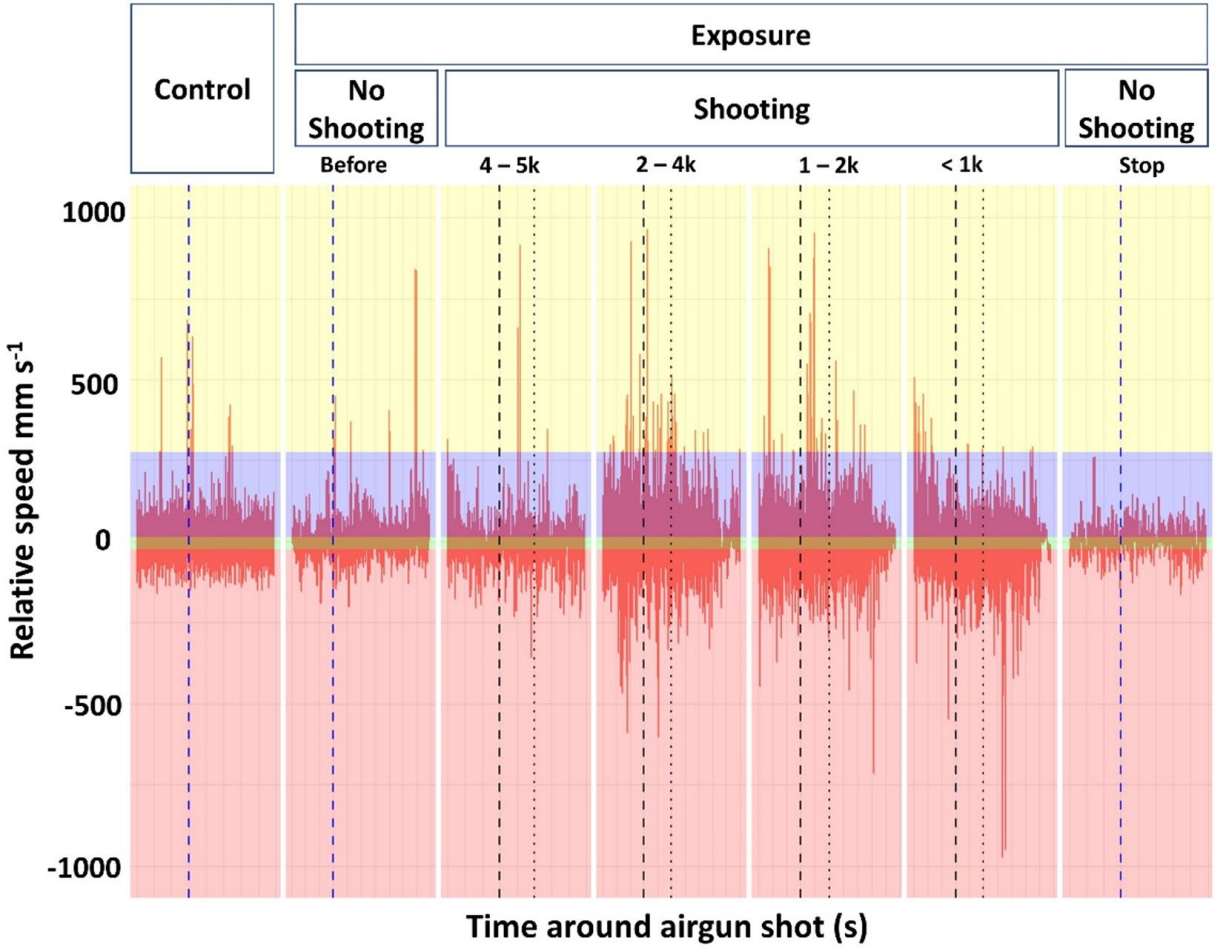
Relative speeds over Distances. The figure shows the relative speeds (y-axis) at the various distances to the approaching airgun array of a commercial seismic vessel (m) and without shooting during the exposure trials over time. The relative time around the airgun amplitude peak, which is set to 0 s (dashed lines), is shown on the x-axis. The vertical black dashed lines indicate the timing of the airgun amplitude peak (time = 0) of the shot. Vertical blue dashed lines represent time 0 for the “No Shooting” and “Control” periods. The vertical dotted line marks 1 s after the airgun peak. The horizontal color distribution shows the relative speed range for the categorized behaviors where red is *Swimming* against currents (< −25 mm s^−1^), green is *Sinking* and *Swimming* (> − 25 and < 14 mm s^−1^), *Swimming* is blue (> 14 and < 274 mm s^−1^), *Jumping* is yellow (> 274).

### Exposure versus control

Overall relative swimming speeds, representing the directionality of the animals’ behaviors exposed to seismic airgun shots were slightly lower but did not significantly different from the control (Fig. 2; GLMM, *n* = 23929, t(4) = −0.666, *p* = 0.506) neither did they in absolute values, the magnitude of activities (GLMM, *n* = 23929, t(5) = 0.368, *p* = 0.713). Maximum swimming speeds were significantly higher in the exposure group (mean 77.2 ± 1.9 mm s^−1^ SEM), showing an estimated (proportional change predicted by the model) increase of 9% compared to the control (26.3 ± 1.5 mm s^−1^; Fig. 2; GLMM, *n* = 4494, t(4) = 3.051, *p* = 0.002). Maximum speeds per individual did not differ between the control (198 ± 26.5 mm s^−1^) and exposure groups (217 ± 12.2 mm s^−1^; GLM, *n* = 223, t(3) = 0.614, *p* = 0.54), but exposed copepods exhibited significantly lower minimum speeds (−181 ± 10.6 mm s^−1^) by estimated 6% in average than the control group (−129 ± 2.70 mm s^−1^; GLM, *n* = 223, t(3) = −2.129, *p* = 0.03).

### With vs. without airgun shots

Maximum relative speeds of the exposure group were significantly lower during non-shooting phases (66 ± 4 mm s^−1^) compared to shooting phases (79 ± 2 mm s^−1^; GLMM, *n* = 2739, t(4) = −3.13, *p* = 0.002; Fig. 2). Individual copepod maximum relative speeds did not differ between non-shooting (193 ± 30 mm s^−1^) and shooting phases (221 ± 13 mm s^−1^; GLM, *n* = 188, t(3) = −0.779, *p* = 0.437), but individual copepods had a lower minimum speed during shooting phases (−194 ± 12 mm s^−1^) compared to non-shooting phases (−101 ± 7 mm s^−1^; GLM, *n* = 188, t(3) = 3.253, *p* = 0.0014).

### Time–exposure and distance interaction effects on maximum speeds

When analyzing the full dataset (including control and exposure groups), the best-fitting model showed a significant interaction between the exposure group and observation time (modeled as a quadratic term), where ‘observation time’ refers to each 4 s subsample reflecting short-term behavior patterns around an airgun shot (GLMM, *n* = 4494, t(6) = 3.694, *p* < 0.001). Zooming into exposure, we tested the effect of distance and observation time (quadratic term) and showed highest maximum speed were at 2000–4000 m distance to the airgun shots (111 ± 4.5 mm s^−1^; GLMM, *n* = 2739, t(12) = 4.350, *p* < 0.001). Here, a slight inverted U-shaped relationship was observed, where maximum speeds were highest around the shot —starting low, increasing, then decreasing again. However, this quadratic interaction with observation time was not statistically significant (GLMM, *n* = 2739, t(12) = −1.152, *p* = 0.249). The only significant interaction revealed that the effect of observation time on swimming speed differed significantly at distances < 1000, showing a positive quadratic relationship (U shape: GLMM, *n* = 2739, t(12) = 2.082, *p* = 0.04).

### Fluid flow speeds

**Fig. 3:**
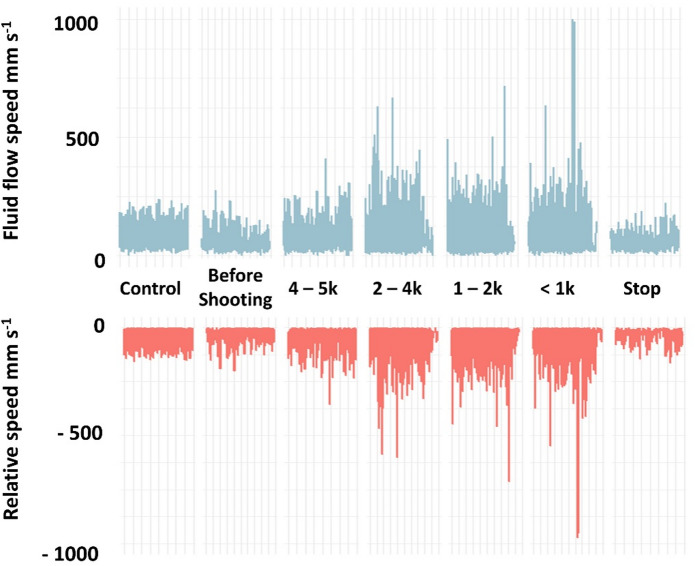
Fluid flow speeds. The x-axis represents the experimental conditions, including control and exposure. Within the exposure category, the phases are before shooting, during shooting (distances to the approaching seismic array (m)), and stop (after shooting). X-axis correspond to observation time (s). The upper y-axis displays the fluid flow speed, while the lower figure’s y-axis shows the relative speeds of the copepods, focusing on values below zero.

Fluid flow speeds did not significantly differ between the control (86 ± 1 mm s^−1^) and the exposure trials (69 ± 0.4 mm s^−1^; GLMM, *n* = 23929, t(4) = −1.414, *p* = 0.157) but were significantly lower before the shooting (47 ± 1 mm s^−1^; GLMM, *n* = 23929, t(9) = −3.679, *p* < 0.001) and during the stop phase (56 ± 1 mm s^−1^; GLMM, *n* = 23929, t(9) = −2.436, *p* = 0.01) compared to the control (86 ± 1 mm s^−1^ see Fig. 3). Maximum fluid flow speeds were higher during the exposure (117 ± 1.5 mm s^−1^) compared to the control (88 ± 1 mm s^−1^; GLMM, *n* = 4494, t(8) = 1.971, *p* = 0.048). Within exposure shooting, fluid flow speeds increased by approximately 3 mm s^−1^ per unit decreasing distance (GLMM, *n* = 19982, t(4) = 11.70, *p* < 0.001; Fig. 3).

Additionally, we tested how fluid flow speeds (mm s^−1^) influences copepod speed-based behaviors (Fig. 2). Fluid flow speeds varied across different speed-based behaviors: The fluid flow speed averaged 115 ± 0.7 mm s⁻¹ while copepods were holding position (i.e., swimming against currents), 50 ± 0.3 mm s⁻¹ during swimming, 58 ± 0.4 mm s⁻¹ while sinking, and 64 ± 4 mm s⁻¹ during jumping. Generalized mixed effect binomial models showed that higher fluid flow speeds significantly decreased the likelihood of movement speeds related to swimming and sinking behaviors (GLMM, *n* = 23915, swimming: z(3) = −63.328, *p* < 0.001, sinking: z(3) = −19.79, *p* < 0.001) while it significantly increased swimming against the currents (GLMM, *n* = 23915, z(3) = 72.730, *p* < 0.001, see Fig. 3). There was no significant effect of fluid flow speeds on the jumping behavior (GLMM, *n* = 23915, z(3) = −1.792, *p* = 0.0731). Median and mean Reynolds (Re) number was 106.6 and 129.6 ± 0.9 (*n* = 23963), respectively. Quantitatively, Re in the control group (*n* = 3955), was on average 151.2 ± 1.1, while during the exposure phase without blasting Re was 100.9 ± 6.1 (Before Shooting; *n* = 2442) and 98.2 ± 2.5 (Stop, *n* = 581). During airgun array shooting, Re ranged from 126.8 ± 1.3 to 131.3 ± 2.1 (*n* = 16985).

### Behavior proportions

**Fig. 4:**
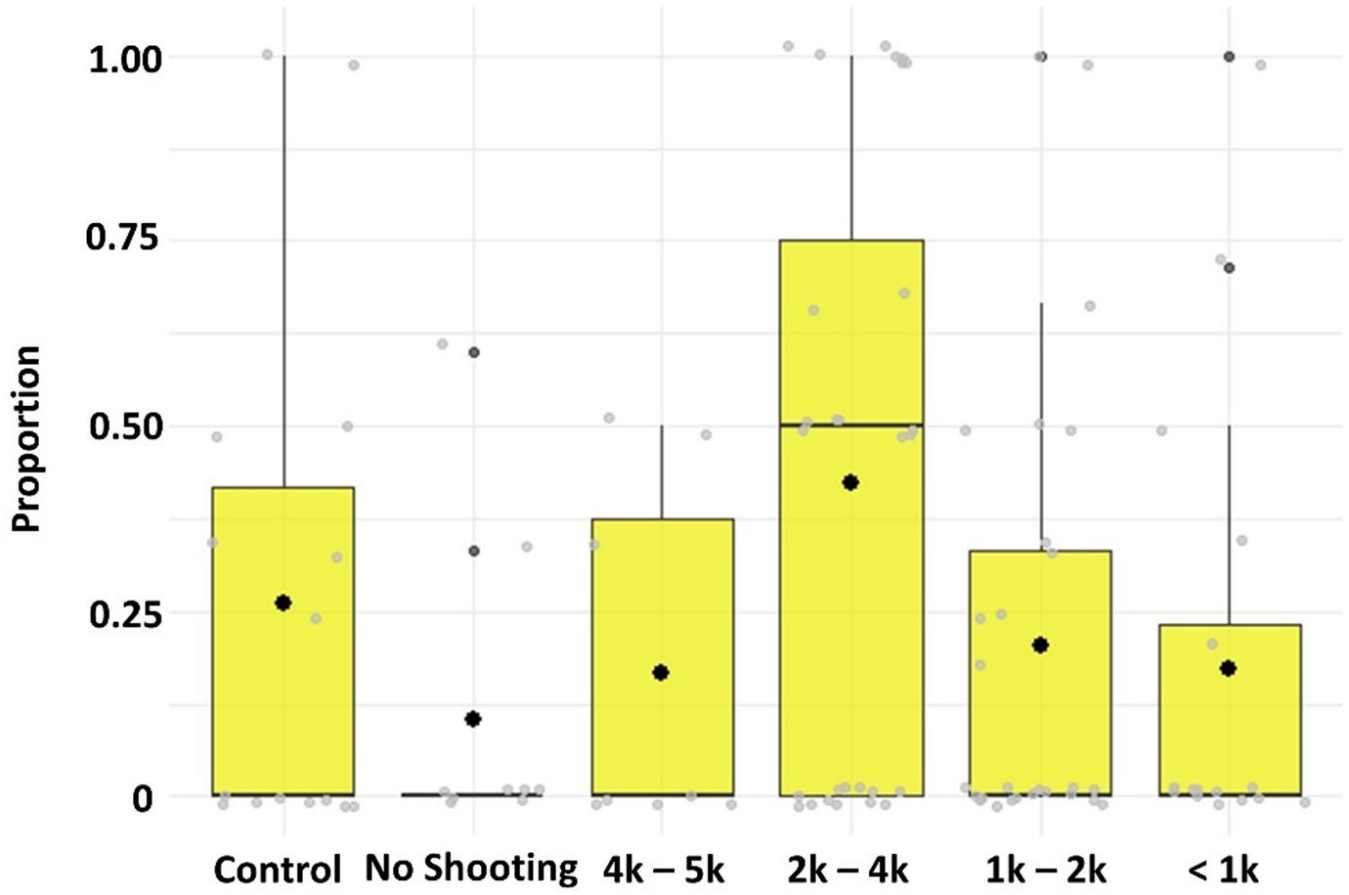
Proportion of copepods that conducted at least one jump. Proportions of the number of copepods that conducted a jump per subsample is shown on the y-axis. X-axis is used to compare proportions between the different exposure conditions (Control *n* = 15, Exposure: No Shooting *n* = 10, and Shooting 4000–5000 m *n* = 10, 2000–4000 m *n* = 28, 1000–2000 m *n* = 27, and < 1000 m *n* = 16 to the approaching airgun array). The boxplots display data from the first to the third quartile, with a black horizontal line indicating the median and a dot the mean. The whiskers extend to the lowest and highest values within 1.5 times the interquartile range. Black dots present outliers and grey dots data per sub sample (*n* = 106).

Based on the relative speeds and angles we counted the occurrences of each behavioral category: *Swimming* (including swimming and swimming against the currents), *Sinking*, and *Jumping*. The categorized behaviors *Swimming* and *Sinking* were observed in nearly all animals (99% (*n* = 220) in each), while *Jumping* was detected in 27% (*n* = 61) of the copepods. A significant increased proportion of copepods exhibited jumping behavior at distances between 2000 and 4000 m from the seismic shooting (ZAGA mu: *n* = 103, t(8) = 2.311, *p* = 0.02; Fig. 4).

### Behavior counts and durations

**Fig. 5:**
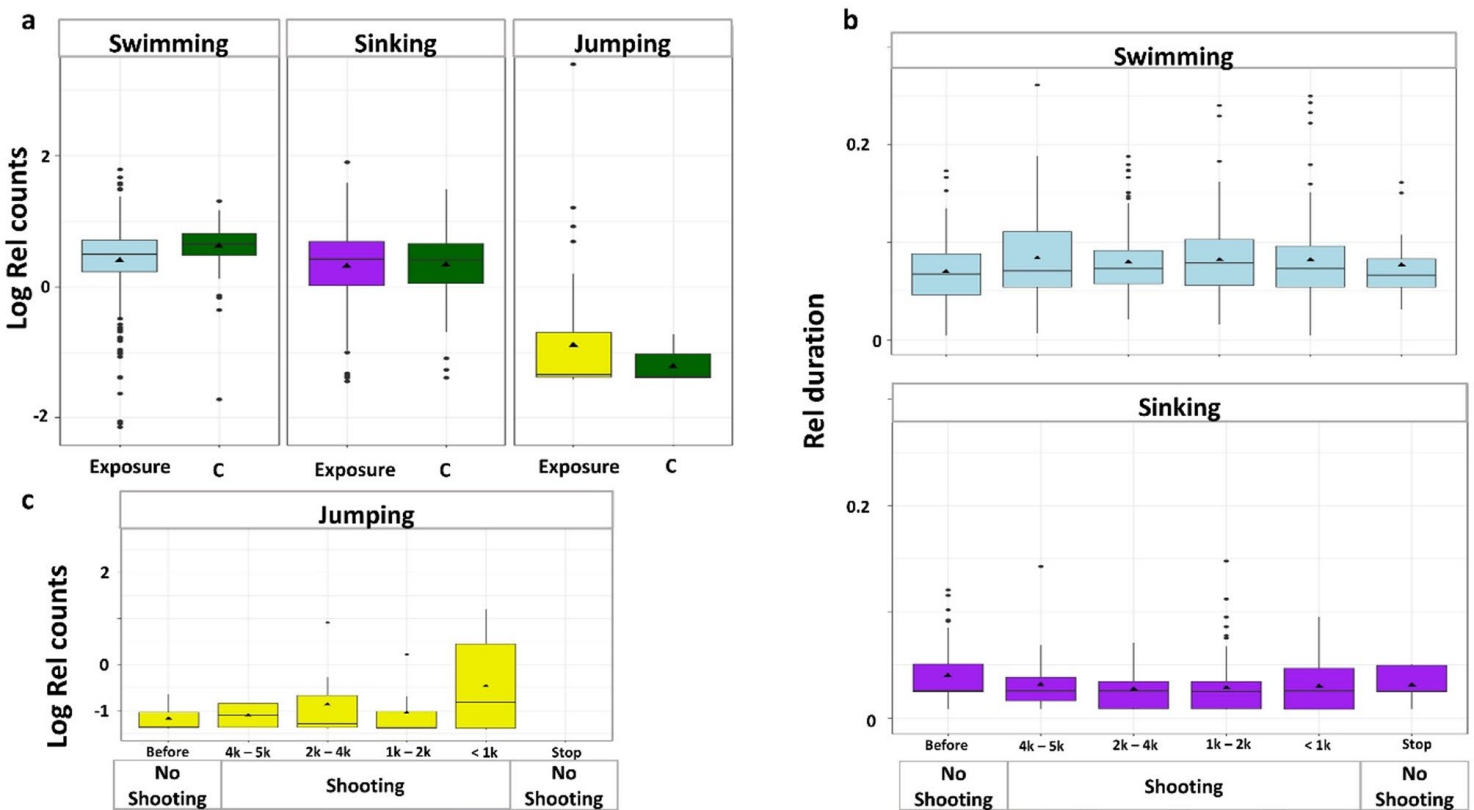
Behavioral counts and durations of each behavior. (**a**) shows differences between behavioral counts per second in the control (n-behaviors = 218, green boxplots) and the exposed group (n-behaviors = 1113, blue (swimming), purple (sinking), and yellow (jumping) boxplots). The (**b**) relative durations per second of swimming and sinking and (**c**) relative jumping counts per second are shown during airgun exposure over the different exposure categories (*n* = 6), including before airgun shooting, distances to the approaching seismic array and after airgun shooting. The boxplots display data from the first to the third quartile, with a black horizontal line indicating the median and the black triangle the mean. The whiskers extend to the lowest and highest values within 1.5 times the interquartile range.

Behaviors showed significant differences in counts (KW, χ²(2) = 173.1, p-value < 0.001) and duration (KW, χ²(2) = 657.3, *p* < 0.001). *Swimming* was performed with a mean relative count (relative to individual observation time) of 1.73 ± 0.03 (median = 1.73; *n* = 633), with a mean relative duration of 0.1 ± 0.0001 (median = 0.09). *Sinking* had a relative count of 1.57 ± 0.03 (median = 1.51; *n* = 622), with a mean duration of 0.03 ± 0.00003 (median = 0.025). *Jumping* occurred with a median relative count of 0.26 (excluding zero counts; mean = 0.86 ± 0.39; *n* = 76) and lasted 0.036 ± 0.004 on average (median = 0.015).

*Jumping* events were significantly fewer by estimated 28.2% during the control trials (0.03 ± 0.009, mean per individual including zeros) compared to the exposure trials (0.1 ± 0.05, mean per individual including zeros; Fig. 5a; ZAGA, *n* = 669, t(64) = −7.847, *p* < 0.001). There was no difference in the counts of *Swimming* (GLMM; *n* = 633, t(5) = 0.621, *p* = 0.534) nor in *Sinking* (GLMM, *n* = 622, t(4) = −0.354, *p* = 0.723) between exposure and control (Fig. 5a). The durations of all three behaviors did not differ between the control trial and the exposure trials (*Swimming* GLMM; *n* = 633, t(5) = −1.144, *p* = 0.253; *Sinking* GLMM; *n* = 622, t(5) = −0.787, *p* = 0.431; *Jumping* GLMM, *n* = 76; t(4) = −0.11, *p* = 0.913).

There was no difference in swimming counts during non-shooting periods compared to shooting periods (GLMM, *n* = 529, t(5) = −1.40, *p* = 0.16) but *Swimming* was shorter in duration during non-shooting periods (GLMM, *n* = 529, t(4) = −2.074, *p* = 0.038). A tendency was found for a quadratic effect of distance on the counts (GLMM, *n* = 529, t(4) = −1.936, *p* = 0.053; Fig. 5b) but not on duration (GLMM, *n* = 531, t(5) = −1.242, *p* = 0.214). There was a non-significant tendency in counts towards more (GLMM, *n* = 519, t(4) = 1.937, *p* = 0.053; Fig. 5b) and significantly longer *Sinking* events in non-shooting compared to shooting periods (GLMM, *n* = 519, t(4) = 2.4, *p* = 0.016). The distance had a significant quadratic relationship on counts (GLMM, *n* = 519, t(4) = 2.324, *p* = 0.02) and on duration (GLMM, *n* = 519, t(4) = 3.107, *p* = 0.0019). Jumping counts per individual were 28.1% less during non-shooting compared to periods with shooting (Fig. 5a, ZAGA, *n* = 564, t(55) = −5.884, *p* < 0.001) and slightly, not significantly, longer in duration (GLMM, *n* = 65, t(4) = −0.907, *p* = 0.364). The effect of distance had a significant quadratic effect on the number of *Jumps* (ZAGA, *n* = 564, t(55) = −3.787, *p* < 0.001; Fig. 5c), indicating that behavior changed with distance in a non-linear manner while durations did not significantly change with distance (GLMM, *n* = 65, t(4) = −0.656, *p* = 0.512).

## Discussion

This study reveals behavioral responses of *Calanus finmarchicus* to airgun shootings from a commercial seismic vessel at distances ranging from 4000 m to under 100 m. While overall swimming speeds did not differ between exposed and unexposed copepods, exposure triggered brief bursts of faster movement, seen in significantly higher maximum speeds. Minimum speeds were lower in exposed individuals, likely due to increased flow during seismic activity, prompting copepods to compensate to maintain position. The highest speeds occurred at 2000–4000 m, while significant exposure-time interactions appeared only below 1000 m.

*Swimming* duration increased, while *Sinking* duration decreased during airgun shooting compared to non-shooting periods (Fig. 5), indicating a shift toward more active movement. This pattern is further supported by the increased frequency of *Jumping* behavior during exposure trials with airgun shooting compared to control trials (Fig. 5). During the exposure trials, jumping behavior occurred more frequently during shooting events (Figs. 4 and 5), with the majority of jumps taking place within 1.2 s following the airgun shot peak at distances between 1000 and 5000 m from the airgun array (Fig. 2, Supp. 1). The relationship between jumping behavior and distance to the seismic source followed a non-linear pattern, indicating that the response varied with distance. Specifically, *Jumping* frequency peaked at 2000–4000 m from the source before declining at greater distances, suggesting a complex interaction between proximity to the source and copepod behavior.

Increasing fluid flow speeds coincided with an increase in swimming against the current (holding position), while sinking and swimming-related speeds decreased during these periods. The observed changes in maximum swimming speeds, along with increased *Jumping* counts and *Swimming* duration during shooting periods, as well as across different distances, may also be influenced by rising low-frequency sound levels (Figs. 2, 3, 4 and 5). We hypothesize that both environmental factors contributed to the observed behavioral responses, with fluid flow speeds primarily affecting swimming behaviors against currents but not *Jumping* (Figs. 2, 3, 4 and 5).

We observed an additional behavior not captured by the speed-based classification method, namely relocation movements or hops where an individual adjusted its body angle using thrust from a single antenna, repositioning itself without a full escape jump. In some species, this type of reorientation often precedes an escape response^35,36^. Reorientations were observed during airgun shooting, not always followed by an escape jump, but were not captured by our speed-based behavioral metrics (pers. obs., see Methods). These visual observations suggest that copepods may perceive and react to acoustic stimuli more frequently than it is detectable through movement speed analyses alone. These types of reactions could be reliably detected with high-resolution tracking technologies (such as described in Fields et al.^21^ which are well suited for capturing fine-scale body orientation changes.

So far, behavioral responses of copepods to airgun exposure have varied among studies. *Acartia sp.* exhibited significantly reduced swimming speeds over a long period of time, while *Calanus sp.* showed similar but only immediate effects following simulated airgun pressure drops (~ 2 bar - similar to around ~ 55 m from a shot of a modeled airgun array of total 2730 in^3^) under laboratory conditions^22^. In contrast, *C. finmarchicus* exhibited no detectable alteration in predator avoidance behavior under laboratory conditions following in situ exposure to airgun shots (SEL 186 dB re 1 µPa^2^s at 25 m^21^). Thus, findings emphasize species specific sensitivities and context-dependent responses of zooplankton to impulsive sounds^5^, likely influenced by body size (and associated Reynolds number), sensory morphology, and physiology, as observed in previous studies^4,5,22^. Small species like *Acartia* spp. may be more reactive compared to larger species due to their operation at lower Reynolds numbers, making them more sensitive to fine-scale hydrodynamic disturbances. Copepods may also experience physical impacts, especially under near-field conditions where seismic airgun shooting generate steep pressure gradients and high acoustic particle velocities. These intense conditions can exert mechanical stress on soft tissues and pressure-sensitive structures such as the lipid-rich oil sacs found in copepods like *C. finmarchicus*, potentially leading to structural failure, or internal damage^22,23^. In our study, we focused on the 1000–2000 μm size fraction, dominated by *C. finmarchicus* (> 90%) at the seismic survey site^30^. While this approach limited size class comparisons, we demonstrate that larger zooplankton can be affected by impulsive noise.

Field studies, despite differing in the extent of mortality reported, indicate similar general behavioral patterns seen in active acoustics. McCauley et al.^4^ observed a ‘hole’ following airgun shooting (SEL 153 dB re 1 µPa^2^ s) that might have resulted from a change in zooplankton orientation or the dispersal of aggregations. Similarly, Vereide et al.^30^ reported that airgun shooting (maximum SEL 182 dB re 1 µPa^2^ s) affected the spread of zooplankton, suggesting that the distribution of individuals within the layer may shift in either direction with decreasing distance to the airgun array. The behavioral changes observed during airgun shooting in this study—such as jumping, longer swimming durations, and reorientation (pers. obs.)—may help explain the broader patterns identified in these field studies.

Fluid speed measurements reflect the physical environment experienced by copepods, as the reference particles were of similar size, capturing fine-scale motions relevant to their ecology. The median Reynolds number (Re = 107) we measured suggests that the background flow is in the upper transitional regime, where inertial forces begin to dominate over viscosity, but both still play a role in a copepods’ environment^37^. Copepods in this environment may experience moderate inertial effects, potentially influencing their swimming strategies. We found that fluid speeds increased during seismic airgun shooting (Fig. 3), likely reflecting enhanced turbulence. Turbulence is well known to influence copepod behavior, with previous studies showing that copepods adjust their swimming activity in response to background flow—increasing active movement as turbulence intensifies^38^. Under such conditions, copepods typically exhibit more frequent jumping behavior^38,39^. However, *C. finmarchicus* has been shown to adjust its swimming trajectory in response to turbulent vortices rather than initiating escape jump^40^, which contrasts with our findings if turbulence alone explains the observed behavior.

The proximity to the shooting airgun array plays a key role in shaping behavioral responses, likely influenced by increased low-frequency sound (Fig. 1) and small-scale turbulences as discussed before (Fig. 3). Impulsive airgun sound signals are followed by increased fluid motion, as measured up to 25 m from the source in previous studies^21^, also observed in our study up to several thousands of meters away from the airgun (Fig. 3), potentially triggering copepod responses. Our data indicate a correlation between increased small-scale turbulences and higher frequencies of swimming against the current, which is energetically costly. Increased energy demands could contribute to delayed mortality, as reported by Vereide et al.^22,23,30^.

Free swimming *C. finmarchicus* exhibit a characteristic swim-and-sink behavior, with short upward swimming sequences followed by passive sinking^41^, which was also observed in our study. This behavior is influenced by developmental stage and seasonal changes^42^, and plays an important role in their vertical migration and foraging strategies, balancing energy expenditure and access to resources^43^. A disruption of a “normal” swimming pattern, such as a shift to a more active swimming behavior as seen in our study, could result in greater energetic demands^38^, and more active copepods may be more “conspicuous” to their fish predators^33,44^. Further, escape jumps in copepods are ~ 400 times more energetically costly than their normal swim-and-sink behavior^45^. Copepods’ energy reserves vary seasonally, being higher in summer and lower in winter due to diapause, so long-term effects may differ by season. Lipid metabolism plays a key role in regulating diapause timing^46^ and since activity levels strongly influence metabolic rates^47^, increased movement during airgun shooting could accelerate lipid depletion, potentially shifting the onset or duration of diapause, which in turn may have cascading effects on survival – and population dynamics. Distinct effects of seismic airgun shooting are, however, complex and species dependent with smaller species potentially being more prone^4,22,30^. In this study, shots were fired at intervals of 10.7 s, whereas modern commercial seismic surveys often employ configurations with regimes that increase the rate of acoustic exposure by more than twofold^48^. Under such conditions, zooplankton are subjected to substantially greater cumulative sound levels, which may amplify energetic demands and stress. The biological impacts observed in this study may represent a conservative scenario, while other full-scale surveys could impose greater energetic stress on zooplankton communities. In commercial seismic surveys, drifting water masses may expose zooplankton to multiple seismic transects. Given that we observed effects at 2–4 km, repeated exposures could further increase energetic costs and amplify sublethal or delayed effects, highlighting the need for future studies on cumulative impacts.

This study could not discern dead from alive individuals – hence we do not have data on mortality. During the same cruise, however, Vereide et al.^30^ reported increased mortality in *C. finmarchicus*(the same cohort of animals used in this study) near the seismic source, after seven days post-exposure. McCauley et al.^4^, hypothesized that zooplankton may not show immediate mortality but could be disabled in their sensory capacity – and hence – decreased fitness and increased predation risk through time – though increased predation risk was excluded in Vereide et al.^30^. Our data (Figs. 2 and 5) further show that jumping ceased following the shooting, and swimming and sinking behaviors returned to levels similar to those seen in the *Control* and *Before Shooting* phases, highlighting that copepod behavior is altered during the shooting periods only. Although the immediate energy costs of escape jumps are modest, the cumulative effects, combined with prolonged periods of elevated oxygen consumption and metabolic rates^49^ during shooting periods, may contribute to (delayed) mortality in copepods.

Copepods, such as *C. finmarchicus*, detect hydrodynamic disturbances through mechanoreceptive setae on their antennae, which allow them to sense signals from predators, prey, and mates^50^. We hypothesize that copepods may also detect pressure fluctuations and/or particle acceleration caused by impulsive sound waves, such as those generated by seismic airguns. Prior research exposed copepods to 2 bar pressure drops that would occur in sounds with peak pressure levels of 226 dB re 1 µPa^22^ and to sound exposure levels of 183 dB re 1 µPa² s^21^. In comparison, sound exposure levels during our cruise ranged from 158 dB re 1 µPa² s to 182 dB re 1 µPa² s (maximum 35 kPa_ptp_^30^; see Fig. 1). The observed behavioral responses in this study occurred at sound pressure levels lower (approximately from 165 dB re 1 µPa²s on and at onset of an increase in the amplitude of frequencies below 1 kHz, see Fig. 1) than those reported in previous behavioral studies. This suggests that even at relatively moderate sound levels, seismic airgun exposure can trigger behavioral changes in copepods. Note that the background SEL in our study primarily stemmed from continuous noise produced by the stationary research vessel. Airgun signals, however, were acoustically and temporally distinct, characterized by rapid pressure changes compared to the low-intensity, continuous vessel noise. Behavioral responses were aligned with these impulses (Fig. 2), suggesting they were the primary trigger for the observed behavioral changes. While both sound types overlapped, this reflects real-world seismic survey conditions where continuous and impulsive noise typically co-occur. Figure 1 demonstrates that low-frequency sound levels (below 1 kHz) increase logarithmically and rise above the background sound dominated by the research vessel at distances where animals exhibited most jumping behavior (Figs. 4 and 5). This observational correlation suggests that low-frequency sound may play a role in triggering behavioral responses. Copepods are known to be sensitive to vibrations to frequencies between 40 and 1000 Hz^50^ and pressure changes^51^ thus potentially (in)directly perceiving sounds emitted from airgun shots.

*C. finmarchicus* are apparently able to perceive airgun shots, as significant changes in their swimming behavior were observed when compared to the control group and across varying distances to the seismic survey. We hypothesize that the impulsive sound and alterations in fluid flow speeds contributed to these behavioral changes – although the exact mechanisms remain unknown. Our findings add to the growing body of evidence that invertebrates are sensitive to anthropogenic noise.

## Methods

**Fig. 6:**
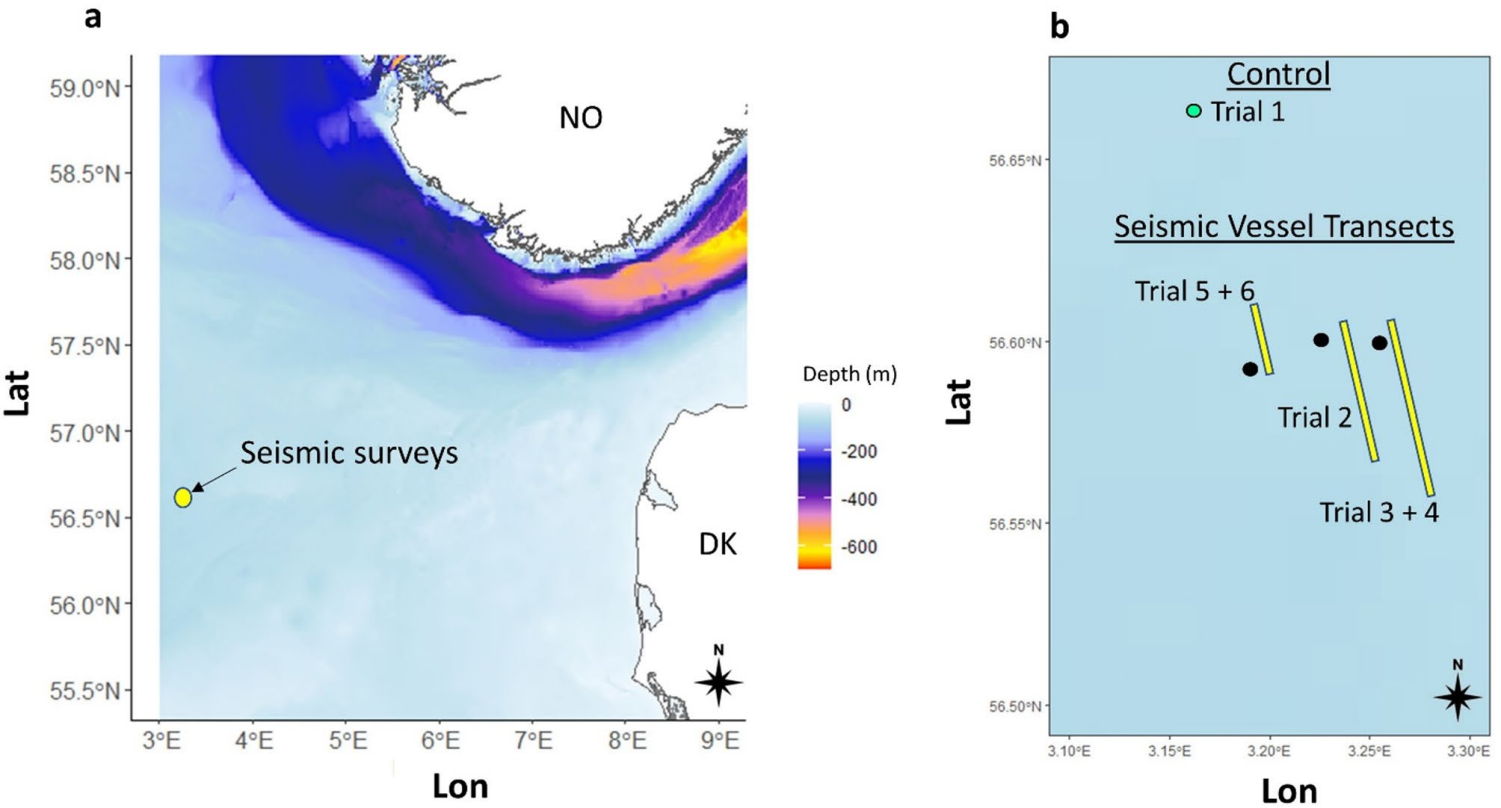
Map of (**a**) the study location and (**b**) seismic vessel transects parts during the trials. The green and black filled dots indicating the position of RV Kristine Bonnevie. The green filled dot (trial 1, 1 st of May) represents the position of the research vessel during one control day where seismic surveys were conducted 15 to 23 km away from the research vessel. The black filled dots represent the position of the research vessel and the yellow lines the transects of the seismic vessel during the trials 2 (2nd of May), 3 + 4 (5th of May), and 5 + 6 (6th of May).

**Fig. 7:**
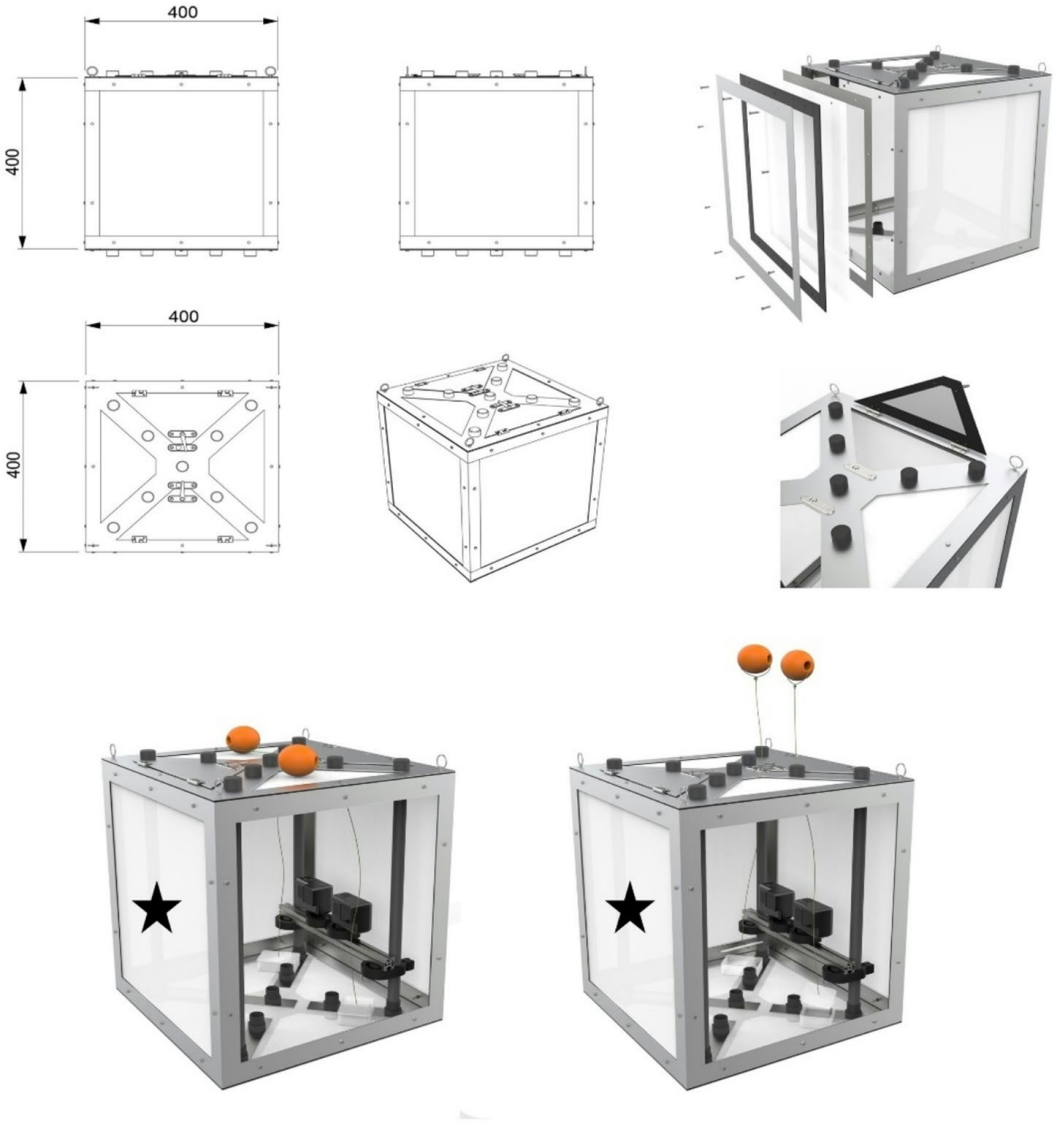
Zooplankton Cage. The cages were constructed using 12 aluminum L-profiles, each measuring 40 cm in length and 3 mm in thickness, welded together to form a cubic structure. The lid and bottom were made from 40 × 40 cm, 3 mm thick aluminum plates, each with 9 holes for threaded pieces and PVC pipes that could be sealed with caps from the outside (bottom and lid). Eye bolts were used on all four corners of the top and bottom to deploy the cage with ropes/buoys/weights. The cage was enclosed with a 100 μm mesh net. Cameras were mounted onto custom-designed 3D-printed camera mounts, attached to a T-rail between two PVC pipes, allowing flexible positioning. On the opposite side of the camera setup (inside the cage)—position indicated with a black star—diving lights and a black background were positioned to provide contrast and illumination for observing copepod behavior. Visualization by Anika Kühn.

The research cruise took place from 29th of April to 8th of May 2022. The following method section describes in situ behavioral experiments with *C. finmarchicus* when exposed to shots from an airgun array (from > 4000 to < 100 m distance). In this study, the research vessel RV Kristine Bonnevie was positioned at a fixed point along a commercial seismic survey transects and at a separate control location outside the transect (Fig. 6b). Zooplankton cages (0.064 m^3^; Fig. 7) equipped with cameras and diving lights were used to observe copepod behavior (up to 2 copepods L^−1^). Video recordings of the experimental trials allowed tracking of individual copepod movements during airgun shooting. Copepod movement trajectories were analyzed to calculate relative speeds in relation to the fluid flow speeds, which were used to categorize different behaviors. Statistical analyses compared relative speeds, behavior counts, and behavior durations between periods with and without airgun shooting, as well as in relation to the distance between the seismic vessel’s airgun array and the research vessel during exposure.

Findings from zooplankton net samples, mortality, mortality experiments, and active acoustics conducted during the cruise aboard RV Kristine Bonnevie are published in Vereide et al.^30^.

### Study area and airgun exposure

Seismic surveys have been conducted since the 1960 s in the Norwegian sector of the North Sea, an oil field that has been operational since the early 1970s^52^ (Fig. 6a). Figure 6 presents the seismic vessel’s transect and the research vessel’s position during 6 experimental trials conducted on the 1 st, 2nd, 5th and 6th of May 2022 (see also Table 1). These transects, combined with the research vessel’s stationary positioning, provided a structured framework to capture copepod behavior across a broad range of distances—from far-field to near-field^4,21^ —using camera deployments optimized for visibility, battery life, and proximity to the airgun array under semi-controlled experimental conditions. On 1st of May, we conducted a control (trial 1; 60 min) during a period when the seismic vessel was shooting at > 15 km away from the research vessel. Trial 2 took place on the 2nd of May for 30 min with the airgun array at a distance of minimum ~ 870 m and maximum ~ 4500 m. Trial 3 and 4 took place on the 5th of May for 54 min with the airgun array at a distance of minimum ~ 90 m and maximum ~ 4460 m. On the 6th of May (Trial 5 and 6), cages were deployed before the onset of the airgun shooting and remained in the water for a total of 46 min. Of this, copepods were exposed to approximately 18 min without airgun shooting, followed by 28 min during periods of airgun shooting. The distance from the airgun array source during this time ranged from approximately 260 m to 1900 m (minimum ~ 260 m and maximum ~ 1900 m). This transect was shorter than the other because only ~ 15 min of position data were recorded during the active airgun shooting period. Positional data from the seismic vessel were only available when the airgun array was shooting and the transect was officially underway. Outside of these shooting periods, the vessel’s location data were not recorded. To estimate the remaining distance of the approach, we assumed the vessel traveled at a speed of approximately 3–5 knots from its last known position.

### Seismic array

Operating at 3–5 knots during a standard commercial survey, the seismic vessel deployed a Sercel G-Gun II airgun array, firing shots every 10.7 s while approaching the stationary research vessel. The source array comprised three sub-arrays (strings), each 8.55 m long and spaced 6 m apart with a total crossline width of 12 m. Each string contained 8 airguns arranged in 4 clusters, operating at 2000 PSI and towed at a depth of 5.0 ± 0.5 m. The full array had a total volume of 3060 in³ (50.1 L, each airgun 127.5 in³ = 2.1 L). Further, each string contained four hydrophones, three depth transducer and one pressure transducer.

### Abiotic conditions

The study area has water depths ranging from 66 to 79 m, increasing westward to approximately 81 m^53^. Details on methods and results related to abiotic conditions are available in Vereide et al.^30^. Briefly, conductivity, temperature, and depth were measured using a CTD (SBE 911plus). Water temperatures in the upper 10 m ranged from 8.4 to 8.5 °C, with salinity consistently between 34.8 and 34.9 PSU. While environmental conditions remained stable on 1 st and 2nd of May, a windy period on 5th of May led to a deepening of the thermocline and pycnocline^30^. These changes, however, occurred at depths below the experimental setup deployments (> 12 m depth). Current speed was between 0.1 and 0.2 m/s, based on model predictions and measurements from a vessel-mounted ADCP (RDI 150 kHz)^54^.

### Experimental animals

*C. finmarchicus* was selected as the experimental crustacean zooplankton species due to its high biomass in the North and Norwegian Sea^55^ and because it supports important fisheries, serving as a valuable prey species for commercially significant fish such as herring, mackerel, and cod^3^.

*C. finmarchicus* (Stage CV - CVI) cultured at SeaLab Norwegian University of Science and Technology (NTNU; Trondheim, NO) were shipped by plane to Bergen (NO) and maintained on board of the research vessel in 20 L buckets (~ 19 copepods L^−1^) filled with 0.2 μm filtered sea water in the dark in a temperature-controlled container at 10 °C and provided aeration until used for experimental trials. Copepod carapace length was 2.5 ± 0.08 mm and antenna span 10 ± 0.06 mm on average.

The same cohort of animals was utilized in Vereide et al.^30^, where the study investigated mortality of in situ sampled zooplankton, their distribution in the water column, and conducted additional mortality experiments using cultured animals. In contrast, our experiment focused on behavioral responses under the same exposure conditions, providing complementary insights.

### Experiments and analysis

#### Observing in situ copepod behavior

We built zooplankton cages to observe in situ copepod behavior during real-life seismic surveys (see Fig. 7 for construction details). Cameras (Models: GoPro Hero 7, GoPro Hero CHDHA-301) mounted 25 cm deep into the cage were positioned 4 cm from each other in the middle of the cage and angled at 10° towards each other. A black matt PVC piece (dimensions: L 30 cm* H 20 cm) served as the background, fixed onto PVC pipes positioned 27 cm away from the camera setup. Diving lights (Volador, LED: 4*Cree XM-LM (U4), Output 360 LM) were used for illumination and to attract the animals into the view of the camera. Animals were maintained (~ 5 min) in small plastic containers (100 mL) with lids connected to floats outside the cage, which opened automatically as the cage descended into the water. These easy-built cages are flexible to use with a variety of small aquatic organisms, camera setups, lights and other sensors that can be mounted into such cages for observing animal behaviour in various environmental situations. Note that the partially open mesh cages allowed some vertical flow, but with ambient currents (see abiotic conditions), any bias from the cage was likely negligible.

#### Experimental procedures

100 to 130 *C. finmarchicus* (1.6 to 2 copepods L^−1^) were selected from the culture and split into two 100 mL containers that were mounted into each cage. The animals were kept in these containers for approximately 5 min until they were released into the water that flooded the cage upon deployment (Fig. 7). One (trial 2) to two cages (trial 3 + 4, and trial 5 + 6) were prepared with the above described equipment (Fig. 7). The two cages were connected by a rope, positioning one cage 2 m above the other. The upper cage was attached to a buoy with a 10 m long rope, while the lower cage was stabilized with a floating device and weights to minimize movement from waves. The cameras had to be turned on before closing the lid on the cage, and the start time was noted for later calculations of the distance between the airgun array and the research vessel. The cage(s) were then lowered into water and remained there until the airgun array passed the research vessel (see Table 1).

#### Sound recordings & analysis

Underwater sound was recorded continuously during three seismic vessel approaches with an Ocean Sonic Eth-X2 hydrophone (sensitivity: 205 dB re 1 µPa, sample rate: 512 kHz; see detailed analysis^30^. The hydrophone was deployed on a steel bracket hanging from a rope from the port side of the research vessel at a depth of 10 m - the same depth as the experimental setup. For this study, representative sound recordings were analyzed from the seismic vessel approach on 5th of May. Specifically, calibrated Sound Exposure Levels (SEL; broadband: 0.1–20 kHz) were calculated using Python (3.12) and visualized in R (ggplot2^56^). Additionally, a spectrogram was generated to illustrate frequency–sound level characteristics at varying distances from the seismic vessel’s airgun array. The function Spectrogram from the Scipy package (1.15.2) was used to calculate the power/Hz over 20 s windows using FFT with a Hamming-window (50% overlap). Power was averaged over 10 Hz-bins and dB was calculated as 10*log (Power). The heatmap was created with Seaborn 0.13.2^57^.

#### Video samples

The following steps were done in Blender (v2.79). Videos (mp4) from the right and the left camera per trial were firstly synchronized based on audio signatures (Supp. 2).

Copepod trajectory sub-samples (samples from one trial, see Table 1) were taken based on airgun shot waveforms, from the camera sound files, that were visually differentiated from the ambient sound as displayed in Supp 2. Here, an airgun shot is characterized by a short-time (~ 0.03 to ~ 0.2 s, observations from wave files video- and hydrophone recordings) increase of the wave amplitude.

Each sub-sample corresponded to one seismic shooting or non-shooting event, consisting of up to 4 s (120 frames) centered on the highest airgun amplitude peak in the video’s soundtrack (Supp. 2). We selected up to 45 frames before and up to 106 frames after the peak to analyse copepod behavior around an airgun array shot. Sub-samples without detectable airgun signals were selected by applying the same time interval structure as used for the shooting samples. The final duration within that range for behavioral analysis was determined based on the visibility time of individuals within the camera’s field of view, which ranged from a total of 105 to 120 frames. The first sub-sample in each trial was collected 5 min after the cage had stabilized in its underwater position, allowing for acclimation time. Subsequently, sub-samples were generated around an airgun array shot, if the following criteria were met: (i) at least one copepod within the field of view, (ii) individuals could be continuously tracked before and after the shot, (iii) ability to identify the copepod in the left and right camera views, and (iv) visibility of the reference particle in both camera views. For each sub-sample, synchronized right and left video frames were saved as JPEG images for copepod trajectory analysis.

**Table 1:** Samples Overview. In total six trials were analyzed from which trial 1 was a control without airgun array shot. The table shows for each trial, information on the timing of the shot, deployments depth (m), distance range (maximum to minimum) from the airgun array, and the number of copepod trajectories analyzed.

Date	Trial	Shooting	Depth (m)	Distance (m)	Trajectories
01.05.2022	**1**	None	10	> 15 000	35
02.05.2022	**2**	At deployment	10	4500 − 870	36
05.05.2022	**3**	At deployment	10	4460–90	32
05.05.2022	**4**	At deployment	12	4460–90	44
06.05.2022	**5**	10 min after deployment	10	1900 − 260	31
06.05.2022	**6**	10 min after deployment	12	1900 − 260	45

#### Tracking copepods

Python (v3.9) was used to track individual copepods’ trajectories. For this, copepods were manually tracked frame by frame (= 105–120 jpgs) for each sub-sample. The tracking process entailed clicking on the midpoint of each copepod’s body frame by frame. In addition to tracking all copepods within the field of view, we identified neutrally buoyant particles, such as Echinodermata larvae, that served as reference points for measuring fluid flow speeds within the cages.

By extracting copepod coordinates from left and right camera views, we were able to triangulate 3D coordinates (x, y, z) of each copepod per frame. To facilitate this process, a checkerboard with dimensions of 7*9 boxes á 20*20 mm was filmed underwater in each cage setup immediately after each trial. This ensured that the checkerboard was visible on left and right cameras for accurate triangulation. In Python, synchronized images from the left and right cameras (waterproof action cameras, 1080p30) of the checkerboard were used to obtain the camera matrices and distortion coefficients for each camera. Following this, a stereo calibration was conducted to derive a rotation matrix and translation vector that accurately represented the transformation between the two cameras. With the camera matrices, rotation, and translation information available, we could use the Direct Linear Transformation algorithm to triangulate the 3D coordinates of points from their corresponding 2D coordinates in stereo image pairs^58^.

#### Speeds and behaviors

The extracted trajectories were then analyzed in R v4.2.1^59^.

Speeds, as referred to throughout the manuscript, were calculated between each pair of consecutive frames by dividing the distance between a copepod’s coordinates at Frame *t* and Frame *t + 1* by the time resolution. The time resolution is the time difference between two consecutive frames, here equaling 1/30 s. The same was done for the fluid flow speed reference.

$$\:Speed\:v=\:\frac{\sqrt{{({x}_{t+1}-{x}_{t})}^{2}+{({y}_{t+1}-{y}_{t})}^{2}+{({z}_{t+1}-{z}_{t})}^{2}}}{Time\:Resolution}$$

The formula contains the calculation of Euclidean Distance (ED) in 3D space. ED was adjusted so that *x*_*t*_, *y*_*t*_, and *z*_*t*​_ represent the coordinates at time t and *t + 1*, *yt + 1*, and *zt + 1*​ represent the coordinates at time t + 1. Secondly, the relative speed to the fluid flow speed reference was calculated (trajectory speed – fluid flow speed reference).

Angles, as referred to throughout the manuscript, were calculated as the angle between the 3D movement vector of the copepod and that of the reference fluid flow, using the dot product formula. The angle at each frame t reflects how closely aligned the copepod’s movement is with the surrounding flow. Both vectors were constructed from the displacement between coordinates at Frame *t* and Frame *t + 1*.

$$\theta={{cos}}^{-1}\left(\frac{{\overrightarrow{v}}_{cop}\cdot{\overrightarrow{v}}_{a}}{{\parallel\overrightarrow{v}}_{cop}\parallel\cdot\parallel\overrightarrow{{v}_{a}}\parallel}\right)*\frac{180}{\pi}$$

θ is angle in degrees, where $\:\overrightarrow{v}$ are the copepod (cop) and fluid displacement (a) vectors between two frames. The dot product captures their directional similarity, and normalization by the vector norms ensures scale invariance.

The relative speed towards the fluid flow speeds individual^−1^ and the movement angle between copepods and the fluid flow were used to categorize behaviors. The behavior categories were: *active Swimming*,* Sinking*,* and Jumping*. Note, that behaviors including the position of the copepod body in space or body angle, for instance “re-positioning” could not be included because this could not be detected by speed solely.

We used relative speeds and angles for identifying behaviors as follows:

$$\:\varDelta\:v=\:{v}_{cop}-{v}_{a},\:\text{a}\text{n}\text{d}\:{\theta\:}_{threshold}$$

where *Δ*v is the speed (v) of copepods (cop) relative to the fluid flow speed (a). Behaviors were first classified based on $\:\varDelta\:v$, and the distribution of their corresponding angles was then analyzed to derive $\:{\theta\:}_{threshold}$ — which is an empirical cutoff for identifying flow-aligned behavior. An angle threshold was applied only to *Sinking* and *Swimming* behaviors, as *Jumping* could be reliably identified based on their distinct speed characteristics using using scalar $\:\varDelta\:v$ alone.

i) Since *sinking* is a passive behavior, copepods are carried by the surrounding fluid and thus move at the same speed as the reference fluid flow, resulting in a relative speed close to zero. Additionally, while sinking, copepods are advected by the surrounding fluid, and their movement direction aligns to some degree with the ambient flow. We can make the rule:

$$\:\varDelta\:{v}_{sink}=if\:{v}_{cop}-{v}_{a}\:\approx\:0\:and\:\theta\:\le\:{\theta\:}_{threshold}$$

ii) *Swimming* copepods will be faster than fluid flow speed reference > 0. However, we observed that some copepods hold their position by actively swimming against the currents. In this case, the speed of the copepod is lower than the fluid flow speed, leading to a relative speed of < 0.

$$\:\varDelta\:{v}_{swim}=\:{v}_{cop}-\:{v}_{a}\:>0,\:and$$

$$\:\varDelta\:{v}_{swim}=\:{v}_{cop}-\:{v}_{a}\:<\:0, and$$

$$\:\varDelta\:{v}_{swim}=\:{v}_{cop}-{v}_{a}\:\approx\:0\:and\:\theta\:>{\theta\:}_{threshold}$$.

iii) Jumping copepods will move faster than the reference particles used to measure micro-scale fluid flow speeds and faster than swimming copepods, resulting in a relative speed greater than that of swimming (+).

$$\:\varDelta\:{v}_{jump}=\:{v}_{cop}-{v}_{a}\:>\left(+\right){v}_{swim}$$

In the next step, we defined when a behavior started and ended. Here, we counted all behavior changes based on the beforehand specified relative speed criteria. If the relative speed remained within the defined behavior range for two consecutive time-steps (0.07 s in total, prior pers. obs.), it was considered as a single behavior. If the speed deviated from that behavior range for more than two consecutive time steps, we registered it as new behavior. We calculated the (i) *relative* number of occurrences of each behavior per copepod as well as the (ii) *relative* duration of each behavior per copepod towards their observation times.

#### Distance calculations

Times between the research vessel, the airgun array of the seismic vessel, and the experimental video recordings were synchronized. Distances between the RV *Kristine Bonnevie* and the airgun array were then calculated for each time step corresponding to an airgun shot, as logged by the seismic vessel. While GPS positions were recorded from the vessel itself, the actual airgun array was towed ~ 111 m astern. To correct for this offset, a time delay of 47 s—based on a vessel speed of 3–5 knots—was applied to estimate when the airgun array would pass the corresponding location. All spatial calculations were performed in R using the *sf* package^60^.

#### Reynolds number

Calculations were done in R v3.4.4^59^.

The Reynolds number (Re) describes the balance between inertial and viscous forces in water. A characteristic length scale of 2.5 mm, representing the approximate body size of *Calanus finmarchicus*, was used to compute this turbulence-related metric.

Re was calculated using the formula:

$$\:Re=\:\frac{p*v*L}{\mu\:}$$

where ρ is the seawater density (~ 1026 kg/m³), v is the flow speed (m/s), L is the characteristic length scale (2.5 mm), and µ is the dynamic viscosity of seawater (~ 1.45 × 10⁻³ Pa·s).

### Statistical analysis

Statistics were performed in R v3.4.4^59^. For data visualization we used the package ggplot2^56^. Means are reported with ± standard error (SEM) unless otherwise stated, and a significance threshold of *p* < 0.05 has been implemented.

For the following analysis, it is important to note that individual copepods were nested within different trials, which may have resulted in repeated measurements. To account for this, we performed prior model selections considering random effects for trials and individuals, both separately and nested (GAMLSS^61^, lme4^62^), as well as models without random effects for all response variables and selected fixed effects presented in the following. Ultimately, the best model was selected based on the lowest AIC, with ΔAIC < 2 having the fewest degrees of freedom^63^. The choice of distribution for each model was based on the response variable’s characteristics. Generally, relative speeds followed a gaussian distribution, while individual maximum and minimum speeds, relative counts (only *Jumping*), and relative durations were better modeled with gamma distributions. This approach ensured that the assumptions of the models aligned with the data’s distributional properties. Finally, only the results from the best model are presented (see Results).

#### Relative speeds

*I. Control versus exposure and exposure: non-shooting versus shooting periods*.

We conducted two separate analyses, each with different fixed factors and response variables. First, we compared relative swimming speeds between the control and exposed groups (fixed factor). Second, we focused on the exposure group, examining differences between shooting and non-shooting periods (fixed factor).

We used the following response variables (all speeds were relative to fluid flow):

1. Relative speeds – capturing the full range of movement dynamics over time to examine moment-to-moment swimming behavior.

Here we distinguished between two types of data transformation in order to interpret over behavioral directionality: full range $\:\varDelta\:v$ and magnitudes of activities: $\:|\varDelta\:v|$ (absolute values). To include full-range swimming speeds in our analysis, we applied a simple shift to the speed variable to ensure all values were positive $\:{\varDelta\:v}_{shifted}=\varDelta\:v+\left|\text{min}\left(\varDelta\:\text{v}\right)\right|+1$.

2. Maximum relative speeds – identifying potential bursts of activity and peak performance.
3. Speed range per individual – assessing the highest and lowest speeds achieved during the observation period to gain insight into overall performance capacity.

*II. Time – Exposure and Distance interaction effects on maximum speeds*.

To determine whether bursts of activity (maximum speeds) were dependent on observation time, we followed the same model selection workflow as described previously. Here, observation time refers to the duration each copepod was individually tracked around an airgun array shot. Specifically, we tested whether a linear or quadratic relationship, along with an interaction term, best described the effect of the categorical fixed factors: Exposure Condition including two levels: Control and exposure; and airgun array proximity including five levels: Non-Shooting Periods and Shooting Periods at distances of 4000–5000 m, 2000–4000 m, 1000–2000 m, and < 1000 m away from the airgun array. The best-fitting model was then selected to report p-values.

*III. Fluid flow*.

We analyzed fluid flow speeds and maximum flow speeds across exposure condition (control and exposure) and distances to the airgun array (continuous fixed effect). Additionally, we used a binomial generalized mixed model to assess whether flow speed influenced the likelihood of specific behaviors: *Swimming*, *Swimming* against currents, *Sinking*, and *Jumping*.

From this point forward, we combined *Swimming* and *Swimming against currents* into a single category. Prior analyses showed no significant differences in their occurrence rates, and biologically, both behaviors represent the same underlying activity: swimming. The distinction was originally made to identify individuals with strong swimming effort to maintain position against strong fluid flow speeds, but this represents a difference in intensity rather than a different behavior. Note that combining these categories also increases the sample size.

#### Behaviors

*I. Behavior proportions*

We calculated the proportion of copepods exhibiting different behaviors: *Swimming*, *Sinking*, and *Jumping*. Since nearly all copepods exhibited swimming and sinking at least once per subsample, these behaviors were considered baseline components of their natural movement (see Discussion) and were therefore not suitable for distinguishing treatment effects, i.e., when a behavior is performed by nearly all individuals across all conditions, proportional comparisons become uninformative due to limited variability between categories. Jumping did not occur in all copepods, making it an informative behavioral metric in this case.

To account for the high number of zero observations, we used a Zero-Adjusted Gamma (ZAGA) model^61^, a type of generalized additive model designed for datasets with excess zeros and positively skewed nonzero values. The ZAGA model allows us to separately model the probability of observing a zero and the distribution of nonzero values. Here, we specifically report mu (µ), which represents the expected mean of the nonzero jumping proportions, modeled using a gamma distribution. We analyzed how µ varied across different exposure categories, including Control, Exposure: No Shooting, and Shooting at different distances from the airgun array (4000–5000 m, 2000–4000 m, 1000–2000 m, and < 1000 m).

*II. Counts and durations*

First, we compared the occurrences and durations between the three categorized behaviors using a Kruskal-Wallis test. Next, we examined the effects of experimental conditions on each behavior separately. Specifically, we tested: (i) differences between control and exposure trials, (ii) differences between non-shooting and shooting phases within exposure trials, and (iii) the effect of distance from the sound source (as a continuous variable). Because Jumping behavior had many zero values, we applied a Zero-Adjusted Gamma (ZAGA) model for that behavior, while other behaviors were modeled using generalized mixed models.

## Supplementary Information

Below is the link to the electronic supplementary material.

Supplementary Material 1

## Data Availability

The data generated during and analyzed during the current study are available from the corresponding author on reasonable request.

## References

[1] Steinberg, D. K. & Landry, M. R. Zooplankton and the ocean carbon cycle. *Annu. Rev. Mar. Sci.* **9**, 413–444 (2017). 10.1146/annurev-marine-010814-015924 27814033

[2] Pinti, J. et al. Model estimates of metazoans’ contributions to the biological carbon pump. *Biogeosciences* **20**, 997–1009 (2023).

[3] Lomartire, S., Marques, J. C. & Gonçalves, A. M. M. The key role of zooplankton in ecosystem services: A perspective of interaction between zooplankton and fish recruitment. *Ecol. Indic.* **129**, 107867. 10.1016/J.ECOLIND.2021.107867 (2021).

[4] McCauley, R. D. et al. Widely used marine seismic survey air gun operations negatively impact zooplankton. *Nat. Ecol. Evol.* **1**10.1038/s41559-017-0195 (2017). 10.1038/s41559-017-0195 28812592

[5] Vereide, E. H. & Kühn, S. Effects of anthropogenic noise on marine zooplankton. In The Effects of Noise on Aquatic Life (eds Popper, A. N. & Hawkins, A. D.) 351–357 (Springer International Publishing, (2023).

[6] Siebert, U. et al. Assessment of potential for masking in marine mammals of the Antarctic exposed to underwater sound from airguns. In *Proceedings of the International Symposium on Marine Mammal Biology* 123–130 (2014).

[7] Slabbekoorn, H. et al. Population-level consequences of seismic surveys on fishes: an interdisciplinary challenge. *Fish. Fish.* **20**, 653–685 (2019).

[8] Handegard, N. O., Tronstad, T. V. & Hovem, J. M. Evaluating the effect of seismic surveys on fish — the efficacy of different exposure metrics to explain disturbance. *Can. J. Fish. Aquat. Sci.* **70**, 1271–1277 (2013).

[9] Hildebrand, J. A. Anthropogenic and natural sources of ambient noise in the ocean. *Mar. Ecol. Prog Ser.* **395**, 5–20 (2009).

[10] Kavanagh, A. S., Nykänen, M., Hunt, W., Richardson, N. & Jessopp, M. J. Seismic surveys reduce cetacean sightings across a large marine ecosystem. *Sci. Rep.* **9**10.1038/s41598-019-55500-4 (2019). 10.1038/s41598-019-55500-4 PMC6915703 31844150

[11] Kunc, H. P., McLaughlin, K. E. & Schmidt, R. Aquatic noise pollution: Implications for individuals, populations, and ecosystems. *Proc. R. Soc. B: Biol. Sci.* 283, 20160839; (2016). 10.1098/rspb.2016.0839 10.1098/rspb.2016.0839 PMC5013761 27534952

[12] Erbe, C., Reichmuth, C., Cunningham, K., Lucke, K. & Dooling, R. Communication masking in marine mammals: A review and research strategy. *Mar. Pollut Bull.* **103**, 15–38 (2016). 26707982 10.1016/j.marpolbul.2015.12.007

[13] Kastelein, R. A. et al. Temporary hearing threshold shift in a harbor porpoise (*Phocoena phocoena*) after exposure to multiple airgun sounds. *J. Acoust. Soc. Am.* **142**, 2430–2442 (2017). 29092610 10.1121/1.5007720

[14] Hubert, J., Campbell, J. A. & Slabbekoorn, H. Effects of seismic airgun playbacks on swimming patterns and behavioural States of Atlantic Cod in a net pen. *Mar. Pollut Bull.* **160**, 111680. 10.1016/j.marpolbul.2020.111680 (2020). 33181953 10.1016/j.marpolbul.2020.111680

[15] Weilgart, L. S. The impacts of anthropogenic ocean noise on cetaceans and implications for management. *Can. J. Zool.* **85**, 1091–1116 (2007).

[16] Slabbekoorn, H. et al. A noisy spring: the impact of globally rising underwater sound levels on fish. *Trends Ecol. Evol.* **25**, 419–427 (2010). 20483503 10.1016/j.tree.2010.04.005

[17] Dähne, M. et al. Effects of pile-driving on harbour porpoises (*Phocoena phocoena*) at the first offshore wind farm in Germany. *Environ. Res. Lett.* **8** https://doi.org/10.1088/1748–9326/8/2/025002 (2013).

[18] Heide-Jørgensen, M. P. et al. Behavioral response study on seismic airgun and vessel exposures in narwhals. *Front. Mar. Sci.* **8**, 658173. 10.3389/fmars.2021.658173 (2021).

[19] McQueen, K. et al. Spawning Atlantic Cod (*Gadus Morhua L*.) exposed to noise from seismic airguns do not abandon their spawning site. *ICES J. Mar. Sci.* **79**, 2697–2708 (2022).

[20] McQueen, K. et al. Behavioural responses of wild, spawning Atlantic Cod (*Gadus Morhua L.)* to seismic airgun exposure. *ICES J. Mar. Sci.* **80**, 1052–1065 (2023).

[21] Fields, D. M. et al. Airgun blasts used in marine seismic surveys have limited effects on mortality, and no sublethal effects on behaviour or gene expression, in the copepod *Calanus Finmarchicus*. *ICES J. Mar. Sci.* **76**, 2033–2044 (2019).

[22] Vereide, E. H., Khodabandeloo, B. & de Jong, K. The copepod *Acartia* Sp. is more sensitive to a rapid pressure drop associated with seismic airguns than *Calanus* Sp. *Mar. Ecol. Prog Ser.* **730**, 15–30 (2024).

[23] Vereide, E. et al. Effects of airgun discharges used in seismic surveys on development and mortality in nauplii of the copepod *Acartia Tonsa*. *Environ. Pollut*. **327**, 121469. 10.1016/j.envpol.2023.121469 (2023). 36963455 10.1016/j.envpol.2023.121469

[24] Barton, A. D. et al. The biogeography of marine plankton traits. *Ecol. Lett.* **16**, 1193–1203 (2013). 10.1111/ele.12063 23360597

[25] Kiørboe, T., Saiz, E. & Viitasalo, M. Prey switching behaviour in the planktonic copepod *Acartia Tonsa*. *Mar. Ecol. Prog Ser.* **143**, 65–75 (1996).

[26] Turner, J. T. The importance of small planktonic copepods and their roles in pelagic marine food webs. *Zool. Stud.* **43**, 255–266 (2004).

[27] Dam, H. G. Evolutionary adaptation of marine zooplankton to global change. *Annu. Rev. Mar. Sci.* **5**, 349–370 (2013). 10.1146/annurev-marine-121211-172229 22809192

[28] Cole, M. et al. Effects of nylon microplastic on feeding, lipid accumulation, and moulting in a Coldwater copepod. *Environ. Sci. Technol.* **53**, 7075–7082 (2019). 31125216 10.1021/acs.est.9b01853 PMC7007202

[29] Candolin, U. & Wong, B. B. *M. Behavioural Responses To a Changing World: Mechanisms and Consequences* (Oxford University Press, 2015).

[30] Vereide, E. H. et al. Zooplankton mortality and distribution around a seismic survey. *Sci. Rep.* **15**, 33907. 10.1038/s41598-025-09465-2 (2025). 41028872 10.1038/s41598-025-09465-2 PMC12484595

[31] Bandara, K., Basedow, S. L., Pedersen, G. & Tverberg, V. Mid-summer vertical behavior of a high-latitude oceanic zooplankton community. *J. Mar. Syst.* **230**, 103733. 10.1016/j.jmarsys.2022.103733 (2022).

[32] Lenz, P. H. & Hartline, D. K. Mechanoreception in crustaceans of the pelagic realm. In Physiological Adaptations To Marine Environments (ed Sebert, P.) 77–90 (CRC, (2014).

[33] Yen, J. & Strickler, J. R. Advertisement and concealment in the plankton: what makes a copepod hydrodynamically conspicuous? *Invertebr Biol.* **115**, 191 (1996).

[34] Fields, D. M. & Yen, J. The escape behavior of marine copepods in response to a quantifiable fluid mechanical disturbance. *J. Plankton Res.* **19**, 1289–1304 (1997).

[35] Buskey, E., Lenz, P. & Hartline, D. Escape behavior of planktonic copepods in response to hydrodynamic disturbances: high speed video analysis. *Mar. Ecol. Prog Ser.* **235**, 135–146 (2002).

[36] Michalec, F. G., Schmitt, F. G., Souissi, S. & Holzner, M. Characterization of intermittency in zooplankton behaviour in turbulence. *Eur. Phys. J.* **38**, 1–7. 10.1140/epje/i2015-15108-2 (2015). 10.1140/epje/i2015-15108-2 26490249

[37] Wadhwa, N., Andersen, A. & Kiørboe, T. Hydrodynamics and energetics of jumping copepod nauplii and copepodids. *J. Exp. Biol.* **217**, 3085–3094 (2014). 24948628 10.1242/jeb.105676

[38] Michalec, F. G., Souissi, S. & Holzner, M. Turbulence triggers vigorous swimming but hinders motion strategy in planktonic copepods. *J. R Soc.* **12**, 20150158. 10.1098/rsif.2015.0158 (2015). 10.1098/rsif.2015.0158 PMC4424694 25904528

[39] Ardeshiri, H., Schmitt, F. G., Souissi, S., Toschi, F. & Calzavarini, E. Copepods encounter rates from a model of escape jump behaviour in turbulence. *J. Plankton Res.* **39**, 878–890 (2017).

[40] Elmi, D., Webster, D. R. & Fields, D. M. Copepod interaction with small-scale, dissipative eddies in turbulence: comparison among three marine species. *Limnol. Oceanogr.* **67**, 1820–1835 (2022).

[41] Bainbridge, R. Underwater observations on the swimming of marine zooplankton. *J. Mar. Biol. Assoc. U K*. **31**, 107–112 (1952).

[42] van Duren, L., van Leeuwen, A. & Videler, J. Swimming behavior of developmental stages of the calanoid copepod *Temora longicornis* at different food concentrations. *Mar. Ecol. Prog Ser.* **126**, 153–161 (1995).

[43] Hirche, H. J. Diapause in the marine copepod, *Calanus finmarchicus*—A review. *Ophelia* **44**, 129–143 (1996).

[44] Almeda, R., Van Someren Grève, H. & Kiørboe, T. Behavior is a major determinant of predation risk in zooplankton. *Ecosphere* **8**, e01668. 10.1002/ecs2.1668 (2017).

[45] van Duren, L. A. & Videler, J. J. Escape from viscosity: the kinematics and hydrodynamics of copepod foraging and escape swimming. *J. Exp. Biol.* **206**, 269–279 (2003). 12477897 10.1242/jeb.00079

[46] Skottene, E. et al. Ø. Lipid metabolism in *Calanus finmarchicus* is sensitive to variations in predation risk and food availability. *Sci. Rep.* 10, 22322; (2020). 10.1038/s41598-020-79165-6 10.1038/s41598-020-79165-6 PMC7749129 33339843

[47] Karlsson, K. & Søreide, J. E. Linking the metabolic rate of individuals to species ecology and life history in key Arctic copepods. *Mar. Biol.* **170**, 156 (2023).

[48] Sarah Lim, S. H. et al. 3D penta-source marine seismic acquisition: an innovative approach to address the sampling challenge. *IOP Conf. Ser. : Earth Environ. Sci.* **1003**, 012018 (2022).

[49] Svetlichny, L. & Obertegger, U. Influence of temperature on swimming performance and respiration rate of the cold-water cyclopoid copepod *Cyclops vicinus*. *J. Therm. Biol.* **109**, 103320. 10.1016/j.jtherbio.2022.103320 (2022). 36195388 10.1016/j.jtherbio.2022.103320

[50] Yen, J., Lenz, P. H., Gassie, D. V. & Hartline, D. K. Mechanoreception in marine copepods: electrophysiological studies on the first antennae. *J. Plankton Res.* **14**, 495–512 (1992).

[51] Yen, J. & Okubo, A. Particle and prey detection by mechanoreceptive copepods: A mathematical analysis. *Hydrobiologia* **480**, 165–173 (2002).

[52] Hester, J. 50 years of Ekofisk. ConocoPhillips Retrieved September 2024: (2019). https://www.conocophillips.com/spiritnow/story/50-years-of-ekofisk/

[53] Phillips Petroleum Company Norway. Ekofisk I disposal: Impact assessment – Environmental and societal impacts. Stavanger. L. Takla (Managing Director), Scandinavian Division. (1999).

[54] Utne-Palm, A. C. et al. Does seismic have an effect on zooplankton? — Field study at Ekofisk with RV Kristine Bonnevie. Havforskningsinstituttet, Toktrapport 2022-9 (2022). Retrieved 2025 : https://www.hi.no/hi/nettrapporter/toktrapport-en-2022-9

[55] Aarflot, J. M., Skjoldal, H. R., Dalpadado, P. & Skern-Mauritzen, M. Contribution of Calanus species to the mesozooplankton biomass in the Barents sea. *ICES J. Mar. Sci.* **75**, 2342–2354 (2018).

[56] Wickham, H. ggplot2: elegant graphics for data analysis. *J. R Stat. Soc. Ser. Stat. Soc.* **174**, 245–246 (2016).

[57] Waskom, M. L. Seaborn: statistical data visualization. *J. Open. Source Softw.* **6**, 3021. 10.21105/joss.03021 (2021).

[58] Hartley, R. & Zisserman, A. *Multiple View Geometry in Computer Vision* (Cambridge University Press, 2004).

[59] R Core Team. *R: A Language and Environment for Statistical Computing* (R Foundation for Statistical Computing, 2021).

[60] Pebesma, E. & Bivand, R. *Spatial Data Science: with Applications in R* (CRC, 2023).

[61] Rigby, R. A., Stasinopoulos, D. M. & Lane, P. W. Generalized additive models for Location, scale and shape. *J. R Stat. Soc. Ser. C Appl. Stat.* **54**, 507–554 (2005).

[62] Bates, D., Mächler, M., Bolker, B. & Walker, S. Fitting linear Mixed-Effects models using lme4. *J. Stat. Softw.* **67**, 1–48 (2015).

[63] Zuur, A. F., Ieno, E. N., Walker, N., Saveliev, A. A. & Smith, G. M. *Mixed Effects Models and Extensions in Ecology with R* (Springer, 2009).

