# Supplementary material for: In situ behavioral responses of crustacean zooplankton to an approaching seismic survey
Source: Sci Rep. 2025 Oct 13;15:35566. doi: 10.1038/s41598-025-20568-8 (PMC12518601; doi:10.1038/s41598-025-20568-8)
Supplement: Supplementary file 1 — Supplementary Material 1 [file 41598_2025_20568_MOESM1_ESM.docx]

**Supplementary Information**

**
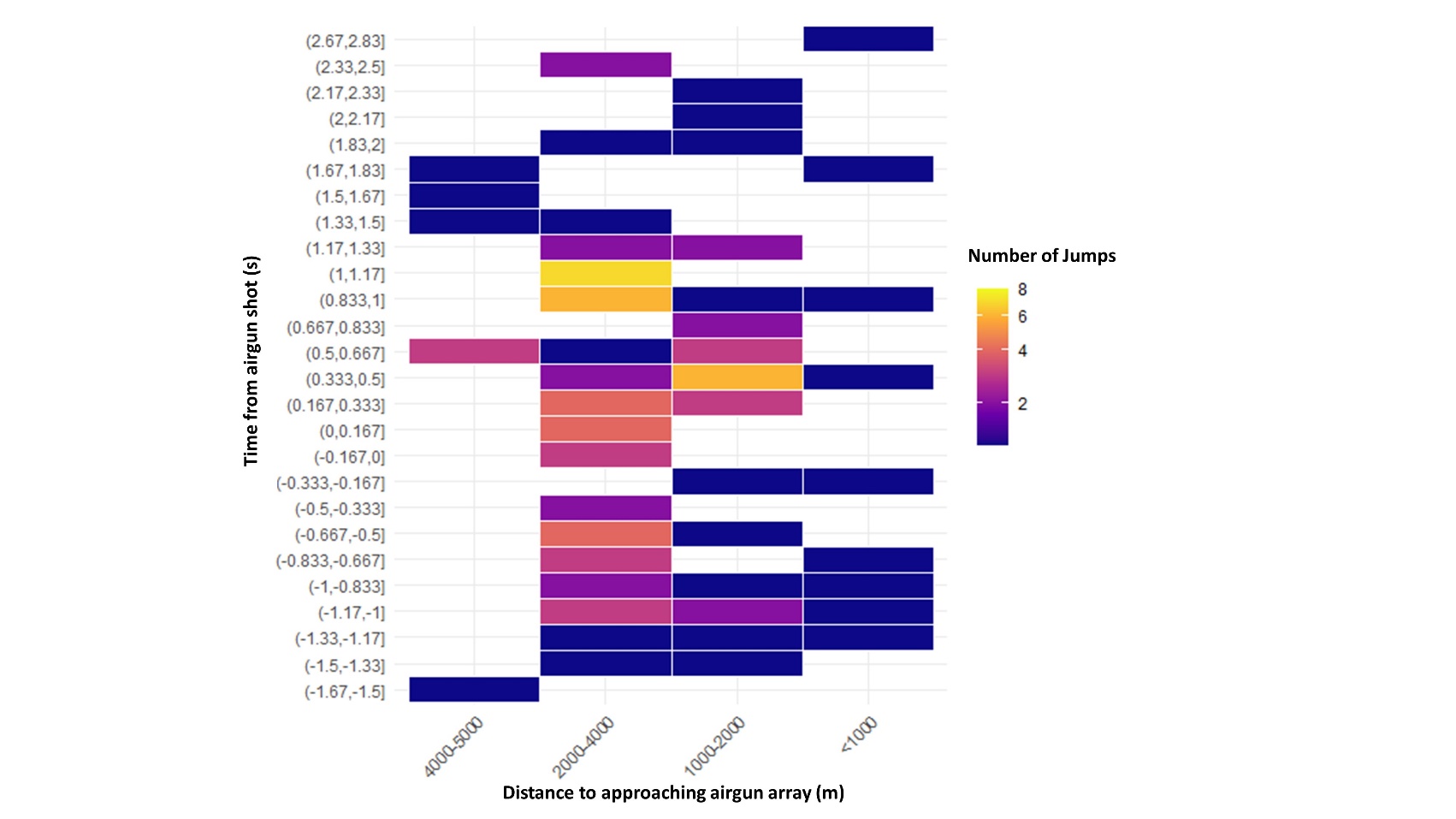
**

**Supp 1: Jumping events during airgun blast exposure**. Y-axis presents the seconds relative to an airgun shot, where the shot occurred at zero. Time bin size is 5/30 seconds. X-axis shows the distance to the airgun array. Color coding represents jumps counted. Only individuals that were present during the airgun signal were included.


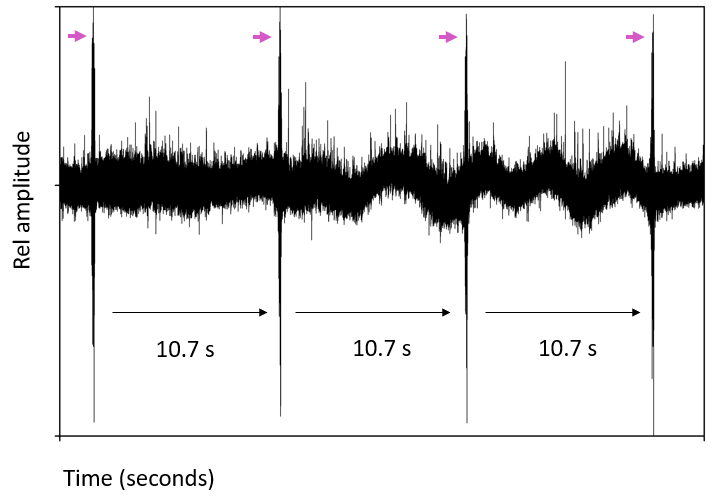


**Supp 2: Airgun Blasts.** Example airgun blast waveform hydrophone recording. The figure represents a waveform from a period of airgun blasts from which sub-samples were taken. The horizontal purple arrows show the airgun blasts as relative amplitude (y-axis) peak. The x-axis shows the time in seconds with 10.7 s between each blast. Note that this is the blast frequency of the seismic vessel. Figure made in Praat (V6.3.09).
